# Kupffer Cells and Peritoneal Macrophage Activation After Platinum Coordination Complexes

**DOI:** 10.1155/MBD.1999.153

**Published:** 1999

**Authors:** Surinder K. Aggarwal, H. J. Muenchen, D. J. Telgenhoff

**Affiliations:** Department of Zoology Michigan State University East Lansing MI 48824-1115 USA


### KUPFFER CELLS AND PERITONEAL MACROPHAGE ACTIVATION AFTER PLATINUM COORDINATION COMPLEXES

Surinder K. Aggarwal*, H.J. Muenchen and D.J. Telgenhoff

Department of Zoology, Michigan State University, East Lansing, MI 48824-1115, USA

**Abstract**

Action of poly-[(*trans*-1,2-diaminocyclohexane)platinum]-carboxyamylose (poly-plat) and cisplatin (CDDP) on the immune system was studied using isolated peritoneal macrophages and compared with liver resident macrophages from normal, CDDP and poly-plat [10 mg/kg] treated Swiss Webster mice for 2-12 days. Peritoneal macrophages were treated [10 mg/L] for 2 h and allowed to grow in normal medium at 37°C (5% $CO_2$) for 24 h. Similarly macrophages from Swiss Webster mice treated with CDDP and poly-plat were isolated and cultured on normal medium. Supernatants from cells in culture were collected at 0.5, 1, 2, and 24 h post-treatment for various cytolytic factors. CDDP and poly-plat treatments induce an activation of the peritoneal macrophages and the Kupffer cells through cell growth, development of cytoplasmic extensions, enhanced number of lysosomes and the activity of various cytolytic factors. Poly-plat treatment of the macrophages induces a 500 fold increase in the number of lysosomes, compared to CDDP treatment where there was only a 50 fold increase.

**"POLY-PLAT"**

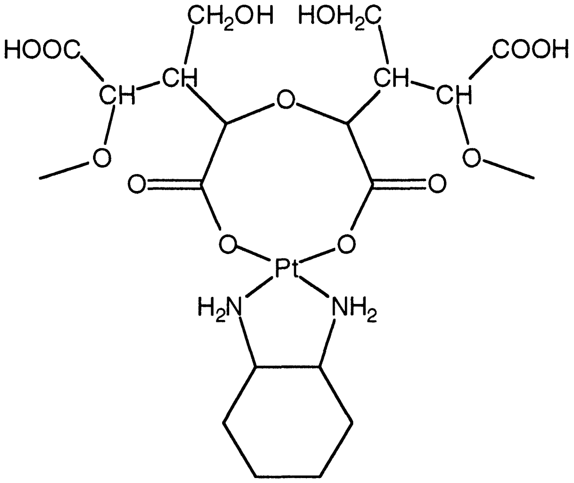

Poly-[(*trans*-1,2-diaminocyclohexane)platinum]-carboxyamylose

### Introduction

Cisplatin (*cis*-diaminedichloroplatinum II), (CDDP) is a broad spectrum antineoplastic agent currently used to treat a variety of cancers [1]. It has been demostrated to inhibit DNA replication and transcription through intrastrand and interstrand crosslinking [2]. It has also been shown to inhibit cytokinesis in cases where the DNA has already replicated through the depolymerization of actin-like microfilaments. Cisplatin has also been shown to enhance specific cellular immune responses in tumor bearing mice [3], through activation of murine peritoneal macrophages [4,5]. Such activated macrophages in culture have been found to destroy tumor cells through the production of various cytolytic factors like interleukin-1α (IL-1α), tumor necrosis factor-α (TNF-α) and nitric oxide (NO) [5,6]. However, it suffers from dose limiting side effects ranging from vomiting, diarrhea, neurotoxicity, nephrotoxicity, and embryotoxicity. These toxicities can be ameliorated through the hydration of patients, by the use of compounds rich in thiols, or the administration of vitamin D [7]. New compounds with differeing ligand are being synthesized in order to increase the efficacy and reduce the toxicities due to cisplatin. Poly-plat (poly-[(*trans*-1,2-diaminocyclohexane)

platinum]-carboxyamylose) is one such compound that has been shown to be more effective in activating macrophages in culture and destroying the target tumor cells through an interaction using lysosomes and various cytolytic factors.

In order to study the effects of poly-plat on the activation of resident tissue macrophages, Kupffer cells comprising the largest population of any fixed tissue macrophage in the body [8] were studied and compared after cisplatin and poly-plat treatments. Morphological characteristics were used to determine Kupffer cell number and size, and these data were compared with light microscopic studies in order to obtain a better understating of both cisplatin's and "Poly-plat's" effects on the Kupffer cell population. Mouse peritoneal macrophages were also used in culture in order to study the release of cytokines from activated macrophages.

**Materials and Methods**

Swiss webster mice (Charles River Laboratories, MA) were sacrificed by cervical dislocation and peritoneal macrophages were isolated according to procedures described earlier [5]. Aspirated cells were seeded onto 18 $mm^2$ glass coverslips, placed in 35 mm petri dishes, at 2-4 x 106 cells/ml and incubated for 2 h at 37°C. Cells were washed vigorously to remove non-adherent cells. Cell cultures were incubated in normal medium (minimal essential media and 10% heat inactivated fetal calf serum) at 37°C in a 5% $CO_2$ incubator. Sarcoma 180 ascites (S180; CCRFS-180II; American Type Culture Collection, MD) served as target cells for macrophages. Normal hepatocytes, obtained by mincing a small piece of liver through a fine wire mesh, 105 x 105 μm in size (Tetko, Inc., IL), also served as target cells for the macrophages. An effector : target cell ratio of 1:10 was maintained in all experiments. Isolated macrophages were treated with poly-plat (5 μg/ml) in 0.85% NaCl for 2 h. The drug containing medium was replaced by normal medium and supernatant (500 μl) was collected at 0.5, 1, 2, and 24 h for cytolytic factor analysis. In addition, macrophages were also treated with cisplatin [5 μg/ml ] dissolved in physiological saline. Untreated cells in normal medium served as controls. For in vivo studies mice (20 g) were injected (ip) with CDDP or poly-plat (10 mg/kg). Mice were sacrificed after 2 or 12 days and peritoneal macrophages were isolated as described above. After 2 h in the culture medium supernatants were analyzed for various cytolytic factors.

Lysosomal assay. The quantification of lysosomes before and after various treatments was achieved by exposing macrophage cultures to fresh medium containing acridine orange (5 μg/ml) for 30 min. at 37°C in the dark [9]. After careful washing macrophages were examined under Zeiss 10 Laser Scanning Confocal Microscope.

TNF-α assay. TNF-α released from supernatants of the macrophages was assayed using specific analysis kits (Genzyme; Cambridge, MA). The multiple antibody sandwich principle was utilized with a murine monoclonal antibody specific for murine TNF-α in the samples [10]. An HRP-conjugated anti-murine TNF-α antibody was used to bind the multiple epitopes on TNF-α. A substrate solution was then added resulting in a color change. The reaction was stopped by acidification and absorbance was read at 450 nm. Standard curves were generated with TNF-α (35-2240 pg/ml) provided in the kits and linear regression analysis was performed.

IL-1α assay. IL-1α was assayed using ELISA kits (Genzyme; Cambridge, MA). The method used the multiple antibody sandwich principle [10], where monoclonal anti-murine IL-1α was used to bind murine IL-1α present in the supernatant. A biotinylated polyclonal antibody binding the IL-1α was added and unbound material was washed out. Peroxidase-conjugated avidin was used to bind these biotin tagged complexes. A substrate solution was then added resulting in a color change. The reaction was stopped by acidification and absorbance was read at 450 nm. Standard curves were generated with IL-1α (15-405 pg/ml) provided in the kits and linear regression analysis was performed.

Inducible Nitric Oxide Synthase (iNOS) Staining. Macrophage monolayers were stained for the inducible enzyme which catalyzes the oxidation of L-arginine to citrulline and NO using avidin-biotin-peroxidase complex method [11]. iNOS was confirmed by VECTASTAIN elite ABC Kit (Vector Laboratories, Inc., Burlingame, CA).

Nitrite Assay for Estimation of Nitric Oxide Production. The concentration of stable nitrite, end product from nitric oxide generation by effector macrophages, was determined by the method of Ding et al [12] based on Greiss reaction. Briefly, 100 μl of supernatant from untreated and treated macrophages collected at various times were mixed with equal volume of Greiss reagent (1% sulfanilamide, 5% phosphoric acid, 0.1% naphthylethylenediamine dihydrochloride; Sigma, MO). The mixtures were incubated for 10 min. at room temperature and the absorbance read at 540 nm. Standard curves were generated using 1 nM-220 μM $NaNO_2$ and nitrite concentrations were determined using linear regression analysis.

For Kupffer cell studies Swiss Webster mice and Wistar rats (Charles Rivers Laboratories, Wilmington MA) were treated with an intraperitoneal injection of either cisplatin (1.8 mg/kg) or polyplat (2 mg/kg) for five consecutive days. On day six animals were killed and liver was excised and freeze sectioned. Sections were fixed in 1% glutaraldehyde, 5% sucrose in 0.5M cacodylate buffer, pH 7.2, for 30-60 seconds at 4° C. Following fixation, the coverslips were washed and stained for nonspecific esterase activity. the population of macrophages in each of the sections was were quantitatively rated based on the number of stained macrophages present within the tissue. These cells were counted per unit area using the same magnification with each section.

For electron microscopy studies variously treated animal liver was perfused with normal saline for one minute, followed by a fifteen minute perfusion fixation with 2% paraformaldehyde in phosphate buffer. The periportal region was excised diced into 1 mm segments and washed in cold PBS. The tissues were then post-fixed in 1% OsO4 in PBS (pH 7.3) for 60 minutes at 4° C, followed by three washings in 0.1 M sodium acetate. The tissues were then washed in a graded acetone series and embedded in araldite. The sectioning was performed on the LKB Ultramicrotome and collected on copper mesh grids, which were then stained with uranyl acetate and lead citrate. The tissues were viewed on the Phillips CM-10 TEM.

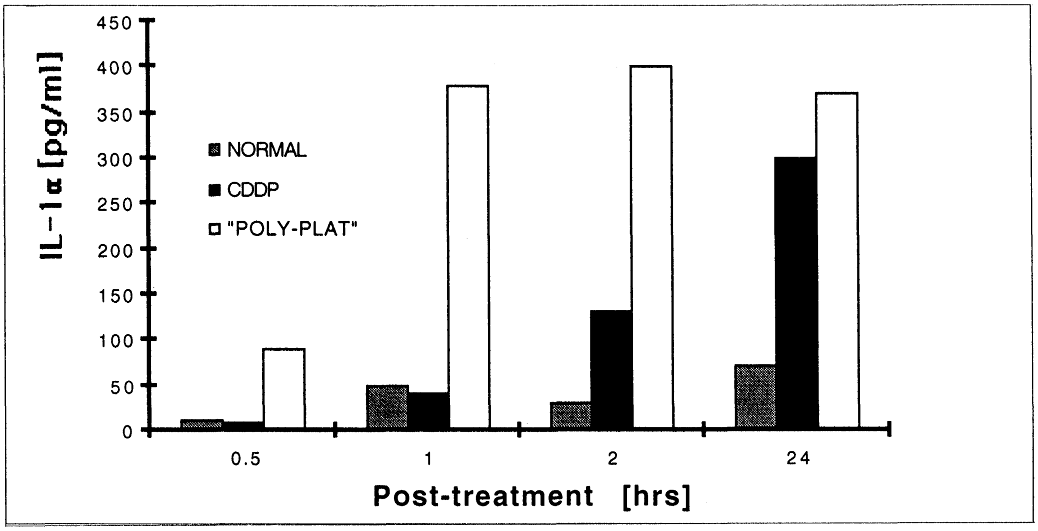

Fig. 1: *In vitro* studies showing the IL-1α release in the supernatants of murine peritoneal macrophages with either CDDP or poly-plat (5 μg/ml) after 30 min, 60 min, 2 h and 24 h post-treatment. Note the large increases in IL-1α levels after 30 min reaching a maximum by 60 min. These levels are maintained for almost 24 h compared to CDDP where the increase is gradual but still less than after poly-plat.

### Results

Normal murine peritoneal macrophages, when treated with poly-plat for 2 h at 37°C in culture, developed extension formations within 2 h radiating from the cell body in all directions. Such extension formation was also observed after cisplatin treatment but only after 24 h. When these poly-plat treated macrophages were co-incubated with S180 tumor cells they immediately established contact with several target cells and formed cytoplasmic continuity through which lysosomes were transported into the tumor cells causing their lysis. CDDP treated cells developed cytoplasmic extensions that were fewer in number and established contact with fewer tumor cells when compared to poly-plat treated macrophages. Normal macrophages did not form cytoplasmic extensions and, when co-incubated with S180 cells, failed to show any interaction for the times of observation.

Lysosomal studies: Based on fluorescence measurements after acridine orange labeling, we observed a 500-fold increase in the number of lysosomes in the macrophages after only 2 h of poly-plat treatment compared to untreated macrophages. The lysosomes were plentiful in the cytoplasm of the macrophages and in the drug-induced cytoplasmic extensions radiating from the cell body. Comparatively, cisplatin treatment demonstrated only a 50-fold increase in the lysosomes, both in the cell body and the cytoplasmic extensions. Poly-plat demonstrated very little TNF-α activity, barely reaching 200 pg/ml at 24 h post-treatment.

Cisplatin demonstrated the usual enhanced release of TNF-α at various time intervals, reaching a peak value at 2 h post-treatment (3000 pg/ml).

Compared to cisplatin treatments there was an increase in IL-1α levels in the supernatants of macrophages treated with poly-plat for up to 24 h of testing. The greatest increases were seen 2 h post-treatment (400-500 pg/ml) with a subsequent decrease from there on (Fig. 1). IL-1α levels demonstrated a consistent increase after cisplatin treatment, reaching a maximum after 24 h. IL-1α levels after poly-plat treatment demonstrated a decline after a peak at 2 h post-treatment but these levels were still equal to or above those after cisplatin treatment. However, macrophages isolated from 'poly-plat' treated mice demonstrated a significantly higher level of IL-1α (500pg/ml) than cisplatin treatment (250 pg/ml), but only after 12 days of the treatment (Fig. 2). Expression of iNOS was visible in poly-plat treated cells while none was detected in untreated macrophages. Only moderate levels of iNOS were visible in cisplatin treated macrophages. Increases in NO levels were detected in cell culture supernatants from poly-plat treated cells at various times. The increase was seen as early as 30 min of the treatment and persisted up to 24 h post-treatment.

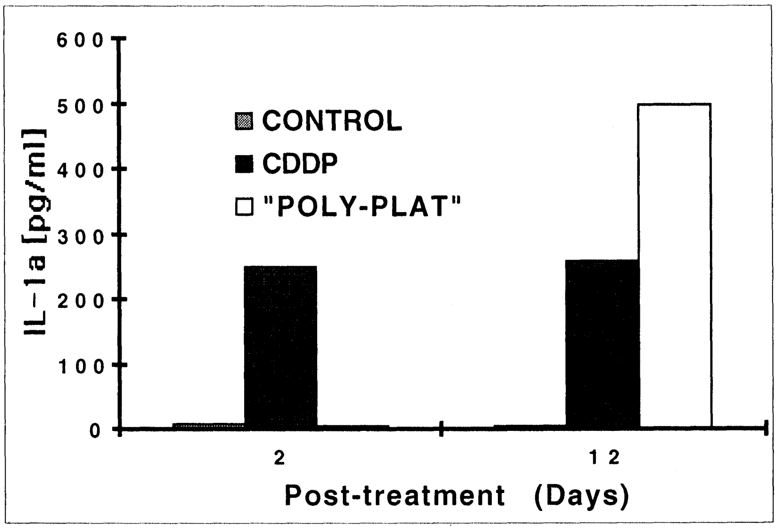

Fig. 2: *In vivo* studies showing the IL-1α release in the supernatants of the murine peritoneal macrophages from CDDP or poly-plat (10 mg/kg) treated mice for 2 or 12 days. Note the interleukin increase in poly-plat treated mice is twice that of CDDP treated animals but only after 12 days.

Cisplatin treatment demonstrated an increase in Kupffer cell extensions. In the normal liver Kupffer cells were round and exhibited few extensions. Poly-plat treatment, however, revealed an even more pronounced activation with a greater increase in extension formation. The normal liver exhibited a moderate amount of esterase staining. The cisplatin treated liver had an increase in esterase staining in the sinusoids, and a significant decrease in the hepatocytes. Poly-plat treatment disclosed a significantly higher degree of staining in the sinusoids than cisplatin, and a moderate amount of staining in the hepatocytes, comparable to the normal liver.

**Discussion**

Stimulation of the immune system is a major function of many antineoplastic agents. Cisplatin stimulates the immune system through macrophage activation making the macrophages more efficient in phagocytosing tumor cells [13]. The severe toxic side effects evident in cisplatin treatment seem to be due to the disruption in sulphydryl containing enzymes, resulting in a decrease in intercellular calcium [14]. Poly-plat shows no such disruption, and as a result has been shown to be much less toxic [15]. Due to the decrease in toxicity poly-plat would appear to be an alternative to cisplatin use. Many agents activate macrophages as well as cisplatin [16,8]. However, there is no significant increase in Kupffer cell numbers recorded after administration of these agents. Poly-plat not only increases Kupffer cell activation, but also increases the number of Kupffer cells present.

The staining pattern observed in light microscopy was the result of the non-specific esterases found in the tissue. The mammalian liver has been shown to exhibit one of the highest concentrations of these esterases compared to other organs, thus making esterase staining a valuable tool for the observation of the various cell types [(17)]. Non-specific esterases catalyze the breakdown of ester bonds, which is a major mechanism of detoxification in the liver. Poly-plat contains ester bonds. Currently it is not known if this drug is active in its natural or biotransformed state. If ester hydrolysis is required for drug activation, then the normal levels of esterase found in poly-plat treated hepatocytes may explain the increased immune system activation seen in Kupffer cells after poly-plat administration as opposed to cisplatin.

The cisplatin treated liver revealed a three-fold increase in Kupffer cells over the control. Self proliferation of Kupffer cells is impossible due to the inhibition of cell division in cisplatin treated tissue [(18)], therefore the increase in numbers are solely the result of monocyte differentiation. These Kupffer cells are more active in phagocytosing invader cells, due to their increase in cytoplasmic extensions [(6)]. These extensions have been shown to seek out and either phagocytose invader cells, or transfer lysosomes down the extensions to lyse invaders [(19)]. Poly-plat does not inhibit cell division [(15)]. The increase in Kupffer cell numbers, however, is too rapid to be caused by macrophage proliferation. Therefore the increase in numbers must be caused by monocyte differentiation as well. Rapidly dividing cells in the bone marrow which are unable to divide during cisplatin treatment can readily produce an increase in circulating monocytes after poly-plat administration, allowing for an even greater immune response over a period of time.

Cisplatin is able to activate peritoneal macrophages *in vitro* and cause lysis of the tumor cells [(4, 13)]. Poly-plat also has the capability to activate macrophages *in vitro*. Our study has shown that poly-plat elicits much more macrophage activation, as depicted by extension formations, lysosomal increases, TNF-α, IL-1α, iNOS, and NO compared to cisplatin. Lysosomes take part in tumor cell death through macrophage activation [(20, 21)]. A greater increase in lysosomes is seen in poly-plat treated macrophages when compared to cisplatin treated macrophages. If lysosomal activity is any indication of cytotoxicity than poly-plat may have greater efficacy in tumor cell death. TNF-α also plays a regulatory role in inflammation and immunological response to tumors [(22)]. TNF-α activates production of nitric oxide which induces iron loss, and inhibits DNA synthesis, mitochondrial respiration and the citric acid cycle [(23, 24)].

IL-1α release by activated macrophages and its cytotoxicity to tumor target cells proves it as a potent mediator in tumor cell killing by macrophages (25). Its release *in vitro* occurs in a cyclic manner showing its greatest increase at 2 h post-treatment after poly-plat. Compared to cisplatin treatment, where the IL-1α release reaches a maximum only after 24 h. The iNOS is induced by a variety of factors including endotoxins (e.g. LPS) and cytokines [IL-1α, IFN-gamma and TNF-α] [(23)]. Results show that poly-plat increases iNOS well above levels reached by cisplatin. NO released by primed macrophages mediates its cytotoxic effects through loss of iron [(26)], inhibition of DNA synthesis, [(28)], and mitochondrial respiration [(27, 29, 30)]. In addition, the reactive oxygen species generated by macrophages could combine with NO to form substances that are more potent than NO itself [(31)]. NO combined with superoxides could yield peroxynitrite that decomposes to hydroxide free radical and $NO_2$ free radical. Our results demonstrate an immediate increase in nitrite concentration in poly-plat treated macrophages after 30 min. in culture. These processes may attribute to the cytotoxic ability of poly-plat primed macrophages inducing the destruction of tumor cells.

### Conclusion

Activated macrophages, efficient in phagocytosing invader cells, are effective scavengers of invading organisms in the bloodstream due to their location in the sinusoid of the liver (Kupffer cells) or in the peritoneal cavity (peritoneal macrophages) [(32, 33)]. Due to the increase in numbers and cytoplasmic extensions, the macrophage populations are better equipped to deal with insult. An increased understanding of the mechanisms controlling macrophage size and number can lead to more effective treatment of distress. These results show that poly-plat is more effective than cisplatin in macrophage activation, with less toxic side effects compared to cisplatin. Further research is needed to determine the active state of poly-plat, and its relation to hepatocellular esterase concentration, as well cytokine production.

**Acknowledgements**

Our sincere thanks to Andrulis Pharmaceuticals for the samples of cisplatin and poly-plat and Dr. Whallon for the use of the confocal facilities.

**References**

1. Sodhi, A. and Aggarwal, S. K. *J Natl Cancer Inst. 53:* 85-101, 1974.
2. Roberts, J. J. and Pascoe, J. M. *Nature. 235:* 282-4, 1972.
3. Bahadur, A., Sarna, S., and Sodhi, A. *Pol J Pharmacol Pharm. 36:* 441-8, 1984.
4. Singh, S. and Sodhi, A. *Exptl Cell Biol. 56:* 1, 1989.
5. Muenchen, H. J. and Aggarwal, S. K. *Anticancer Drugs. 9:* 93-9, 1998.
6. Palma, J. P. and Aggarwal, S. K. *Anticancer Drugs. 6:* 311-6, 1995.
7. Meara, D. J., Johnson, B., Wang, Y., and Aggarwal, S. K. *Anticancer Drugs. 8:* 988-99, 1997.
8. Xu, Z. L., Bucana, C. D., and Fidler, I. J. *Am J Pathol. 117:* 372-9, 1984.
9. Poole, A. *In:* J. Dingle (ed.) *Lysosomes: a laboratory handbook*, pp. 313. Amsterdam: Elsevier/North Holland Biomedical Press, 1977.
10. Meager, A. *In:* F. Balkwill (ed.) *Cytokines: a practical approach*, pp. 299. Oxford: Oxford University Press, 1991.
11. Hsu, S. M., Raine, L., and Fanger, H. *J Histochem Cytochem. 29:* 577-80, 1981.
12. Ding, A. H., Nathan, C. F., and Stuehr, D. J. *J Immunol. 141:* 2407-12, 1988.
13. Palma, J. P., Aggarwal, S. K., and Jiwa, A. *Anticancer Drugs. 3:* 665-76, 1992.
14. Aggarwal, S. K. *J Histochem Cytochem. 41:* 1053-73, 1993.
15. Fiebig, H., Dress, M., Ruhnau, T., Misra, H., Andrulis, P., and Hendriks, H. *Proceedings of the American Association of Cancer Research. 37:* 297, 1996.
16. Sweet, M. J. and Hume, D. A. *J Leukoc Biol. 60:* 8-26, 1996.
17. Johnson, B. N. and Aggarwal, S. K. *In: Microscopy and Microanalysis* 1996, Minneapolis, MN, 1996 1996, pp. 788-789.
18. Rosenberg, B., Van Camp, L., and Krigas, T. *Nature. 205:* 698-699, 1965.
19. Muenchen, H. J., Aggarwal, S. K., Misra, H. K., and Andrulis, P. J. *Anti-Cancer Drugs. 8:* 323-328, 1997.
20. Bucana, C., Hoyer, L., and Hobbs, B. *Cancer Res. 36:* 4444, 1976.
21. Hibbs, J. *Science. 148:* 468, 1974.
22. Balkwill, F., Naylor, M., and Malik, S. *Europ J Cancer. 26:* 641, 1990.
23. Esumi, H. and Tannenbaum, S. R. *Cancer Res. 54:* 297-301, 1994.
24. Knowles, R. G. and Moncada, S. *Biochem J. 298:* 249-58, 1994.
25. Palma, J. P. and Aggarwal, S. K. *Anticancer Drugs. 5:* 615-22, 1994.
26. Hibbs, J., Taintor, R., and Vavrin, Z. *Science. 235:* 473, 1984.
27. Stuer, D. and Nathan, C. *J Exptl Med. 169:* 1543, 1989.
28. Krahenbuhl, J. and Remington, J. *J Immunol. 113:* 507, 1974.
29. Granger, D. and Lehninger, A. J Cell Biol. *95:* 527, 1982.
30. Drapier, J. C. and Hibbs, J. B., Jr. *J Immunol. 140:* 2829-38, 1988.
31. Lowenstein, C. J. and Snyder, S. H. *Cel l 70:* 705-7, 1992.
32. Gupta, P. and Sodhi, A. *Int J Immunopharmacol. 9:* 385-8, 1987.
33. Wisse, E. *J Ultrastruct Res. 46:* 499-520, 1974.

**Received: April 9, 1999 - Accepted: May 3, 1999 -**
**Received in revised camera-ready format: May 4, 1999**

